# Longitudinal Change in CD86 Expression Is Associated with Regression of Cervical Intraepithelial Neoplasia

**DOI:** 10.3390/biomedicines14071456

**Published:** 2026-06-26

**Authors:** Rina Kawatake, Saki Kamata, Risa Yoshida, Rie Maruyama, Naoko Tomita, Yuki Katoh, Hanano Ando-Kobayashi, Nobuki Hayashi, Yuki Okuma, Osamu Kobayashi, Shinichiro Yabe, Keisuke Saito, Yoko Nakanishi, Shinobu Masuda, Kei Kawana

**Affiliations:** 1 Department of Obstetrics and Gynecology, Nihon University School of Medicine, Itabashi, Tokyo 173-8610, Japan; 2 Department of Functional Morphology, Nihon University School of Medicine, Itabashi, Tokyo 173-8610, Japan; 3 Division of Oncologic Pathology, Department of Pathology and Microbiology, Nihon University School of Medicine, Itabashi, Tokyo 173-8610, Japan

**Keywords:** CD86, cervical intraepithelial neoplasia, longitudinal change, biomarker, immune microenvironment

## Abstract

**Background/Objectives**: Cervical intraepithelial neoplasia (CIN) exhibits heterogeneous clinical behavior, with some lesions regressing spontaneously, whereas others persist or progress to higher-grade disease. Identifying biomarkers that reflect lesion dynamics remains a major clinical challenge. This study aimed to evaluate the clinical significance of CD86 expression in cervical lesions by examining longitudinal changes and determining whether temporal alterations in CD86 expression are associated with lesion regression and epithelial-associated immune dynamics. **Methods**: Cervical samples were collected from patients with CIN, and gene expression was analyzed using reverse transcription–quantitative PCR. Longitudinal analyses were performed using paired samples to evaluate the temporal changes in *CD86* expression. Regression status and time to regression were assessed, and associations with *CD86* changes were evaluated using receiver operating characteristic analysis, logistic regression, and Cox proportional hazards models. Longitudinal patterns were further characterized using a spaghetti plot and slope analyses. **Results**: Baseline *CD86* expression did not associate with regression status or CIN grade. However, longitudinal changes in *CD86* expression differed significantly between the regression and non-regression group. *CD86* change demonstrated moderate predictive performance for regression and was significantly associated with both regression and shorter time to regression. Longitudinal analyses revealed distinct temporal patterns between the regression and progression groups. Baseline *CD86* expression was strongly correlated with *FOXP3* expression, whereas *CD86* dynamics were not independently associated with lymphocyte-related markers. **Conclusions**: Longitudinal changes in *CD86* expression are significantly associated with lesion regression in CIN and may reflect lesion-associated immune dynamics during follow-up, particularly within epithelial-derived cervical cytology specimens.

## 1. Introduction

Cervical cancer is one of the most common malignancies in women, with approximately 600,000 new cases and 340,000 deaths annually worldwide [1,2]. In Japan, both the incidence and mortality of cervical cancer continue to rise, with approximately 10,000 new cases and 3000 deaths per year [3]. Cervical intraepithelial neoplasia (CIN) is a precancerous lesion of the cervix. Although many CIN lesions regress spontaneously, some persist or progress to invasive cancer. Approximately 70% of CIN1, 50–60% of CIN2, and 10–20% of CIN3 lesions regress spontaneously [4,5]. Lesion behavior is strongly influenced by the type and persistence of human papillomavirus (HPV) infection [6]. Specifically, HPV-negative CIN2 lesions show high regression rates, whereas persistent HPV16 infection is associated with reduced regression [7,8]. These findings highlight the need for improved risk stratification in CIN management.

CD86, a member of the B7 family, is mainly expressed on antigen-presenting cells and serves as a ligand for CD28 and CTLA4 on T cells [4,9]. CD86 is rapidly upregulated upon inflammatory stimulation, whereas CD80 shows slower induction [4]. In the cervix, CD86 is expressed in epithelial keratinocytes [10]. Reduced CD86 expression is observed in HPV-positive low-grade lesions [10], suggesting a potential role in lesion behavior. Furthermore, CD86 expression has also been reported in non-hematopoietic epithelial cells in other tissues, supporting a broader role in local immune regulation [11].

Accordingly, this study aimed to evaluate the clinical significance of *CD86* expression with a focus on longitudinal changes in cervical lesions. Specifically, we investigated whether temporal changes in *CD86* expression were associated with lesion regression and reflected epithelial-associated immune dynamics. Our findings may help improve understanding of cervical lesion regression by linking longitudinal CD86 changes to lesion-associated immune dynamics in cervical cytology.

## 2. Materials and Methods

### 2.1. Patients and Specimen Collection

This study was approved by the Clinical Ethics Committee of the Nihon University School of Medicine, Itabashi Hospital (approval number: RK-240611-4). Written informed consent was obtained from all participants.

Cervical samples were obtained from 128 patients diagnosed with CIN at the Gynecology Outpatient Department of our hospital (CIN1, *n* = 61; CIN2, *n* = 37; CIN3, *n* = 30). Samples were collected using a cervical brush and preserved in liquid-based cytology (LBC) medium. Cervical cytology specimens predominantly consisted of epithelial cell components (Supplementary Figure S1).

### 2.2. Clinical Outcome Definitions

Clinical outcomes were defined based on cytological and histological findings during follow-up. Regression was defined as follows: for CIN1, two consecutive cytology results negative for intraepithelial lesion or malignancy; for CIN2, histological downgrade to CIN1; and for CIN3, two consecutive cytology results of low-grade squamous intraepithelial lesion or less. Progression was defined as progression from CIN1 to CIN2/3 or from CIN2 to CIN3. Cases that did not meet these criteria were classified as having no change.

### 2.3. HPV Genotyping

HPV genotyping was performed after DNA extraction. The L1 region was amplified using the PGMY09/11 primer set, and genotyping was conducted using PCR-based methods following the standard protocols [12].

### 2.4. RNA Extraction and cDNA Synthesis

Total RNA was extracted from LBC-stored scrapings using an miRNeasy Mini Kit (QIAGEN GmbH, Hilden, Germany). cDNA was synthesized using the High-Capacity cDNA Reverse Transcription Kit (Applied Biosystems, Thermo Fisher Scientific, Waltham, MA, USA) following the manufacturer’s protocol.

### 2.5. Reverse Transcription–Quantitative PCR (RT-qPCR)

RT-qPCR was performed to measure gene expression in cervical samples. *GAPDH* was used as an internal control. Reactions were performed on a QuantStudio 3 Real-Time PCR System using TaqMan Universal PCR Master Mix (both Thermo Fisher Scientific) following the manufacturer’s instructions. TaqMan Gene Expression Assays (Applied Biosystems, Thermo Fisher Scientific) were used for *CD80* (Hs01045161_m1), *CD86* (Hs01567026_m1), and *FOXP3* (Hs01085834_m1). *CTLA4* expression was analyzed using the PrimeTime qPCR assay (Integrated DNA Technologies, Coralville, IA, USA; assay ID: Hs.PT.58.3907580). All samples were analyzed in triplicate. Relative gene expressions are presented as either ΔCt or 2^−ΔCt^ values, where ΔCt represents the difference between the Ct value of the target gene and that of *GAPDH*. Statistical analyses were performed using ΔCt values.

### 2.6. Immunohistochemistry (IHC)

IHC was performed using formalin-fixed, paraffin-embedded tissue sections (4 μm). After antigen retrieval and blocking, sections were incubated with primary antibodies against CD86 (BLR030F, 1:200, Abcam, Cambridge, UK), CD68 (#76437, 1:400, Cell Signaling Technology, Danvers, MA, USA), and CD80 (EPR1157(2), 1:5000, Abcam, Cambridge, UK) overnight at 4 °C. Detection was performed using an appropriate secondary antibody system, followed by visualization with 3,3′-diaminobenzidine and hematoxylin counterstaining. To provide supportive quantitative assessment of the representative staining patterns, CD86 and CD80 staining were semi-quantitatively evaluated using the H-score. FOXP3 staining was evaluated as the percentage of FOXP3-positive cells [13].

### 2.7. Statistical Analysis

Statistical analyses were performed using GraphPad Prism (version 11; GraphPad Software, San Diego, CA, USA) and R software (version 4.2.1). Continuous variables were analyzed using the Mann–Whitney U test (for two groups). Categorical variables were analyzed using Fisher’s exact test.

Correlations between variables were assessed using Spearman’s rank correlation coefficient. Receiver operating characteristic (ROC) curve analysis was performed to evaluate the predictive performance of *CD86* change, and the optimal cut-off value was determined using the Youden index.

Logistic regression analysis was used to evaluate associations with regression status. Cox proportional hazards regression analysis was performed to assess the factors associated with time to regression. Both univariate and multivariate analyses were conducted, and odds ratios (ORs) and hazard ratios (HRs) with 95% confidence intervals (CIs) were calculated.

Kaplan–Meier curves were constructed to estimate cumulative regression rates, and differences between groups were compared using the log-rank test. Patients were stratified into high and low groups based on the median *CD86* change, as no established biological threshold exists.

For longitudinal analysis, spaghetti plots were generated, and individual slopes were calculated to quantify temporal changes [14]. Differences in slope values among groups were assessed using the Kruskal–Wallis test, followed by Dunn’s multiple comparison test.

All tests were two-sided, and *p* < 0.05 was considered statistically significant.

## 3. Results

### 3.1. Participant Selection and Study Design

**Figure 1:**
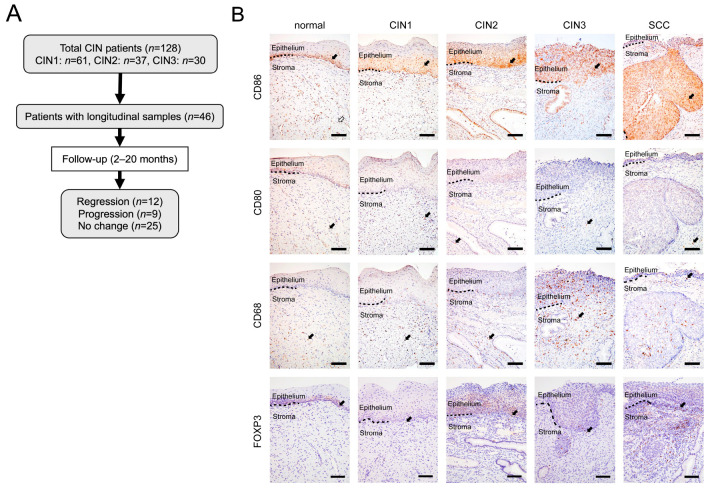
Study flow diagram and immunohistochemical staining for CD86 in cervical epithelium. (**A**) Flow diagram of the patients included in the longitudinal analysis. The study enrolled 128 patients with cervical intraepithelial neoplasia (CIN1, *n* = 61; CIN2, *n* = 37; CIN3, *n* = 30). Of these, 46 patients with paired cervical samples were included in the longitudinal analysis. Patients were followed for 2–20 months and categorized into regression (*n* = 12), progression (*n* = 9), or no change (*n* = 25) groups based on clinical outcomes; (**B**) Representative immunohistochemical staining for CD86, CD80, CD68, and FOXP3 in the normal cervix, CIN grades 1–3, and cervical squamous cell carcinoma (SCC) tissues. The dashed line indicates the epithelium-stroma junction, and the arrows indicate representative positive cells. Scale bars = 100 μm. CIN, Cervical intraepithelial neoplasia.

Of 128 patients with CIN, 46 had paired samples available for longitudinal analysis (Figure 1A). Patients were followed for 2–20 months and categorized into regression (*n* = 12), progression (*n* = 9), or no change (*n* = 25) groups. The baseline clinicopathological characteristics of the study population are summarized in Table 1.

### 3.2. CD86 and CD80 Show Distinct Expression Patterns in Cervical Epithelium

Immunohistochemical staining was performed to evaluate CD86, CD80, and CD68 expression in normal cervical epithelium, CIN grades 1–3, and cervical squamous cell carcinoma tissues. CD86-positive cells were observed within the cervical epithelium, predominantly in the basal layer of normal tissue, with broader distribution in CIN and carcinoma. CD80-positive cells were not detected in the epithelial compartment in any specimen.

CD68-positive cells were primarily localized in the subepithelial stroma, showing a distribution pattern distinct from that of CD86. Notably, CD86-positive cells, but not CD80, were detectable in cervical epithelial cells, suggesting their potential involvement in the epithelial microenvironment. FOXP3-positive lymphocytes were predominantly localized in the subepithelial/basal regions (Figure 1B). Representative negative control staining is shown in Supplementary Figure S2. Representative low- and high-magnification images are shown in Supplementary Figure S3. Semi-quantitative assessment of staining intensity was also performed and is summarized in Supplementary Table S1.

### 3.3. Association of Longitudinal Change in CD86 Expression with CIN Regression

**Figure 2:**
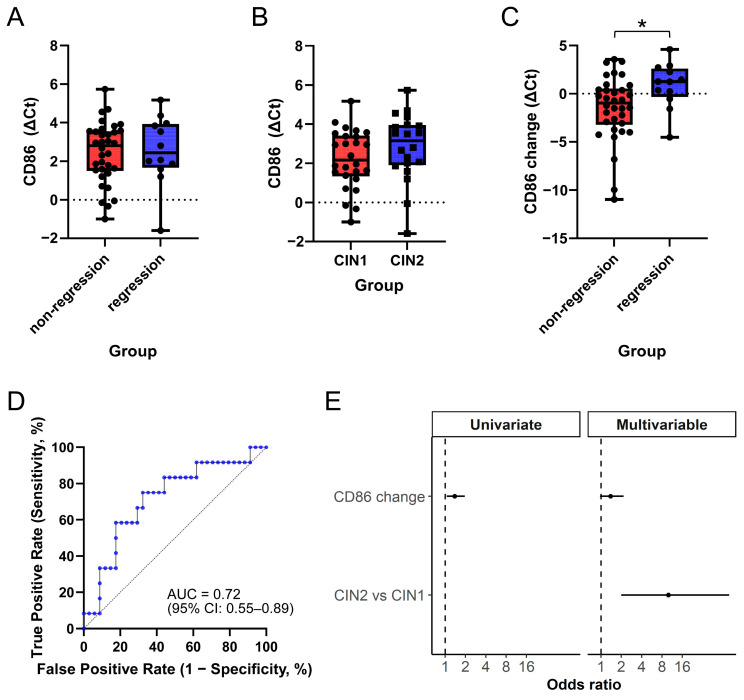
Association of longitudinal change in *CD86* expression with CIN regression. (**A**) Baseline *CD86* expression in the regression and non-regression groups (Mann–Whitney U test); (**B**) Baseline *CD86* expression according to CIN grade (CIN1 vs. CIN2; Mann–Whitney U test); (**C**) Longitudinal change in *CD86* expression in the regression and non-regression groups (Mann–Whitney U test); (**D**) Receiver operating characteristic (ROC) curve analysis of *CD86* change for predicting regression; area under the curve (AUC) is shown. (**E**) Logistic regression analysis evaluating the association between *CD86* change and regression (univariate and multivariate models). *CD86* expression in cervical samples was measured using RT-qPCR and analyzed as ΔCt values. *CD86* change was calculated as the difference in ΔCt values between the latest available time point and baseline for each individual (ΔCt_latest − ΔCt_baseline). In panels (**A**–**C**), dots represent individual samples, and horizontal dotted lines indicate ΔCt = 0. In panel (**D**), the diagonal dashed line indicates the reference line for no discrimination. In panel E, circles and horizontal lines indicate odds ratios and 95% confidence intervals, respectively, and vertical dashed lines indicate an odds ratio of 1. * *p* < 0.05. CIN, Cervical intraepithelial neoplasia; RT-qPCR, Reverse transcription–quantitative PCR.

Baseline *CD86* expression did not differ significantly between the regression and non-regression groups (non-regression: progression + no change) (*p* = 0.67, Figure 2A). Similarly, baseline *CD86* expression did not significantly associate with CIN grade (CIN1 vs. CIN2, *p* = 0.12, Figure 2B).

In contrast, the increase in ΔCt values over time, corresponding to reduced *CD86* expression, was significantly greater in the regression group than in the non-regression group (*p* = 0.025, Figure 2C). ROC analysis demonstrated a moderate predictive ability of *CD86* change for regression (area under the curve = 0.72, 95% CI: 0.55–0.89, *p* = 0.026, Figure 2D). The optimal cut-off value determined using the Youden index was 0.156, yielding a sensitivity of 75% and specificity of 67.6%.

In univariate logistic regression analysis, *CD86* change was significantly associated with regression (OR = 1.38, 95% CI: 1.05–1.96, *p* = 0.039, Figure 2E). In multivariable logistic regression analysis adjusted for CIN grade, the effect size remained similar (OR = 1.38), although statistical significance was attenuated (95% CI: 0.97–2.16, *p* = 0.11). Firth’s penalized logistic regression yielded comparable results, further supporting the robustness of this association.

### 3.4. Longitudinal Dynamics of CD86 Expression and Its Association with Time to Regression

**Figure 3:**
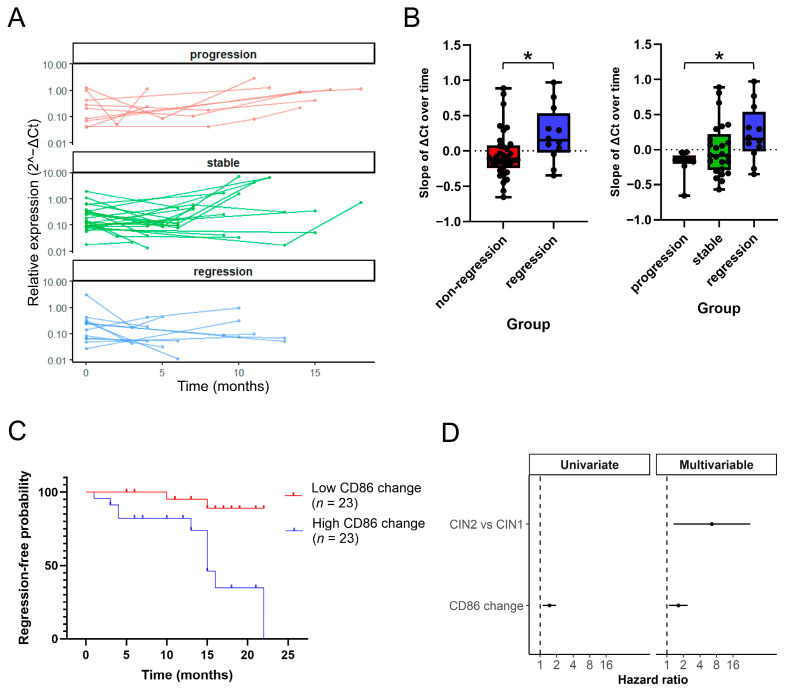
Longitudinal dynamics of *CD86* expression and its association with time to regression. (**A**) Spaghetti plot showing longitudinal *CD86* expression in individual patients across clinical groups (progression, stable, and regression); (**B**) Comparison of the slope values of *CD86* expression. Left: comparison between the non-regression and regression groups (Mann–Whitney U test). Right: comparison among progression, stable, and regression groups (Kruskal–Wallis test followed by Dunn’s multiple comparison test); (**C**) Kaplan–Meier analysis of the time to regression stratified by *CD86* change (log-rank test); (**D**) Cox proportional hazards analysis evaluating the association between *CD86* change and time to regression (univariate and multivariate models). *CD86* expression was measured using RT-qPCR and analyzed as ΔCt values. *CD86* change was calculated as the difference in ΔCt values between the latest available time point and baseline for each individual (ΔCt_latest − ΔCt_baseline). Slope values were calculated from longitudinal measurements for each individual. Patients were stratified into high and low groups based on the median value of *CD86* change for Kaplan–Meier analysis. In panel (**B**), horizontal dotted lines indicate a slope value of 0. In panel (**C**), tick marks indicate censored observations. In panel (**D**), vertical dashed lines indicate a hazard ratio of 1. * *p* < 0.05.

Spaghetti plot analysis demonstrated distinct temporal patterns of *CD86* expression across clinical groups (Figure 3A). The progression group exhibited a general increasing trend over time, whereas the regression group demonstrated a decreasing trend. The stable group displayed intermediate and heterogeneous patterns.

Quantitative analysis of slope values revealed significantly lower slopes in the regression group than in the non-regression group (*p* = 0.013, Figure 3B). When analyzed across the three groups, a significant difference was observed (Kruskal–Wallis *p* = 0.033). Post hoc analysis using Dunn’s test showed a significant difference between the progression and regression groups (*p* = 0.017), whereas the difference between the stable and regression groups showed a trend (*p* = 0.056), and no significant difference was observed between the progression and stable groups (*p* = 0.13).

Kaplan–Meier analysis demonstrated that greater changes in *CD86* expression were associated with significantly shorter time to regression compared with smaller changes (log-rank *p* = 0.001, Figure 3C).

In Cox proportional hazards analysis, *CD86* change was significantly associated with time to regression in both univariate (HR = 1.47, 95% CI: 1.12–1.93, *p* = 0.005) and multivariable models adjusting for CIN grade (HR = 1.62, 95% CI: 1.09–2.40, *p* = 0.017), indicating that greater *CD86* change was associated with faster regression (Figure 3D).

### 3.5. Association of CD86 with the Cervical Immune Microenvironment

**Figure 4:**
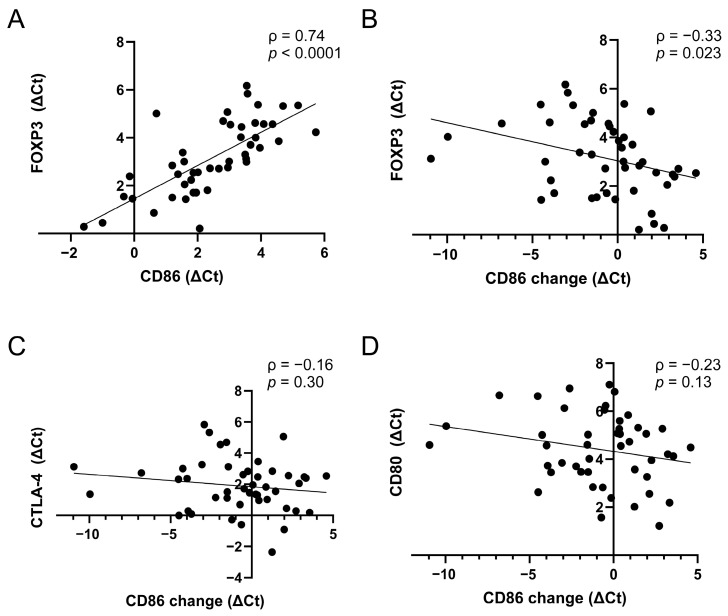
Association of *CD86* expression with the cervical immune microenvironment. (**A**) Correlation between baseline *CD86* and *FOXP3* expression (Spearman’s rank correlation); (**B**) Correlation between *CD86* change and *FOXP3* expression (Spearman’s rank correlation); (**C**) Correlation between *CD86* change and *CTLA4* expression (Spearman’s rank correlation); (**D**) Correlation between *CD86* change and *CD80* expression (Spearman’s rank correlation). Gene expression in cervical samples was measured using RT-qPCR and analyzed as ΔCt values. *CD86* change was calculated as the difference in ΔCt values between the latest available time point and baseline for each individual (ΔCt_latest − ΔCt_baseline). Correlation coefficients (ρ) and corresponding *p* values are shown. Partial correlation analysis adjusted for baseline CD86 expression was performed for *CD86* change and *FOXP3.* RT-qPCR, Reverse transcription–quantitative PCR.

A strong positive correlation was observed between baseline *CD86* and *FOXP3* expression (ρ = 0.74, *p* < 0.0001; Figure 4A), suggesting that *CD86* expression reflects an immune-active microenvironment. In contrast, *CD86* change showed a modest inverse correlation with *FOXP3* expression (ρ = −0.33, *p* = 0.023; Figure 4B). However, this association was attenuated after adjustment for baseline *CD86* expression (partial ρ = −0.25, *p* = 0.10), suggesting that the correlation may partly reflect baseline expression levels rather than an independent relationship. No significant correlations were observed between *CD86* changes and other immune-related markers, including *CTLA4* and *CD80* (Figure 4C,D). Therefore, dynamic changes in *CD86* expression are not fully explained by the lymphocyte-associated markers assessed here.

## 4. Discussion

This study revealed that longitudinal changes in *CD86* expression, rather than baseline levels, were associated with the clinical course of CIN. Specifically, changes in *CD86* expression were consistently associated with lesion regression across multiple analytical frameworks, including ROC analysis, logistic regression, Cox proportional hazards models, and longitudinal slope analyses. Thus, longitudinal assessment of *CD86* expression may provide additional information regarding lesion behavior compared with single time-point measurements.

Although baseline *CD86* expression was not associated with regression status or CIN grade, *CD86* change was associated with both regression and time to regression, suggesting that temporal dynamics more accurately reflect disease behavior than single time-point measurements. The spaghetti plot and slope analyses further supported this observation, demonstrating distinct longitudinal patterns between the regression and non-regression groups. Hence, *CD86* changes may reflect lesion-associated immune dynamics in CIN.

From a biological perspective, *CD86* expression may reflect the local cervical immune environment. Notably, baseline *CD86* expression was strongly correlated with *FOXP3* expression, suggesting an association with an immune-regulatory microenvironment. This observation is biologically plausible, as CD86 preferentially supports the proliferation, survival, and maintenance of FOXP3-positive regulatory T cells [15]. However, the relationship between longitudinal *CD86* changes and *FOXP3* expression was modest and not independent of baseline expression, with no clear associations observed with other immune-related markers. Therefore, although *CD86* expression may reflect the immune-regulatory status of cervical lesions, the mechanisms linking longitudinal *CD86* changes to lesion regression remain unclear. CD86 dynamics may thus represent broader alterations in the local microenvironment rather than changes in a specific immune cell subset. Moreover, representative immunohistochemical staining in this study showed differences in the distribution of FOXP3-positive and CD68-positive cells across lesion grades. Although the present study was not designed to evaluate stage-specific immune cell dynamics, these observations may reflect changes in the local immune microenvironment during CIN progression and warrant further investigation.

A key feature of this study is the use of cervical cytology specimens, which are predominantly composed of epithelial cells, unlike previous studies based on immunohistochemistry or peripheral blood analyses that primarily reflect immune cell-dominant compartments [16,17]. Consequently, *CD86* expression in this context may capture epithelial-associated immune dynamics. As cervical cytology specimens are routinely collected during CIN surveillance, longitudinal assessment of *CD86* expression could potentially be incorporated into existing follow-up strategies without requiring additional invasive procedures.

Predicting lesion behavior remains a major clinical challenge in CIN management. Although HPV genotype and histological grade are established baseline factors, local immune responses also play a critical role in determining outcomes. Increase in intraepithelial lymphocytes is associated with spontaneous CIN regression, whereas higher regulatory T cell levels are linked to persistence, consistent with a balance between effector and immunosuppressive responses governing lesion fate [18]. Our findings suggest that *CD86* changes may serve as a marker reflecting immune microenvironmental dynamics during follow-up rather than a single time-point predictor. Although changes in CD86 expression were associated with lesion regression in univariate analyses, this association was attenuated after adjustment for CIN grade. Therefore, the independent predictive value of CD86 dynamics beyond established clinical factors remains to be clarified in larger studies.

CD86 has been identified as a predictive biomarker of therapeutic HPV vaccine response, with lower expression associated with improved outcomes [19]. Our group previously showed that the oral HPV16 E7 vaccine IGMKK16E7 induced regression of CIN2/3 lesions (MILACLE study) [20,21,22]; however, these findings reflect treatment-induced responses rather than the natural history of CIN. In contrast, the present study focused on patients undergoing routine clinical follow-up without therapeutic HPV vaccination and demonstrated that longitudinal changes in *CD86* expression are associated with spontaneous lesion regression. Thus, *CD86* dynamics may capture intrinsic microenvironmental changes independent of vaccination.

This study also has some limitations. First, gene expression was analyzed in total cervical cell populations containing both epithelial and immune cell components, preventing cell type-specific distinction. As cervical cytology specimens are predominantly composed of epithelial cells, lymphocyte-associated signals may have been relatively underrepresented. Second, the limited number of paired samples may have constrained statistical power, particularly in multivariable models. In addition, the predictive performance of CD86 change was evaluated in a relatively small cohort, and external validation in larger independent populations is necessary before its clinical application. Third, sampling variability inherent to cervical cytology may have influenced the results. Moreover, although HPV genotype is a major determinant of CIN behavior, adjustment for HPV persistence and detailed genotype-specific analyses were not feasible due to the limited number of regression cases and incomplete HPV persistence data. Therefore, HPV-related factors may have acted as potential confounders in this study. Finally, this study did not address the mechanistic role of CD86, and further investigation is warranted to clarify the underlying biological processes.

## 5. Conclusions

Longitudinal changes in *CD86* expression are significantly associated with lesion regression in CIN and may serve as a molecular indicator reflecting epithelial-associated immune dynamics. These findings suggest that longitudinal CD86 dynamics may provide additional information on lesion behavior during CIN follow-up, although validation in larger cohorts is essential.

## Figures and Tables

**Table 1:** Baseline clinicopathological characteristics of the study population. Baseline characteristics of patients with cervical intraepithelial neoplasia (CIN) included in this study. Continuous variables are presented as median (interquartile range), and categorical variables are presented as number (percentage). Comparisons between groups were performed using the Mann–Whitney U test for continuous variables and Fisher’s exact test for categorical variables, as appropriate. HPV genotyping was unavailable in six cases; therefore, percentages may not total 100%. IQR, Interquartile range.

		Regression (*n* = 12)	Non-Regression (*n* = 34)	*p*-Value
Age (years, median [IQR])		32 (28–37)	32 (29–37)	0.84
Follow-up duration (months, median [IQR])		5.5 (3–10)	9 (5–10)	0.24
Parity, *n* (%)		3 (25.0%)	11 (32.4%)	0.73
Smoking, *n* (%)		1 (8.3%)	3 (8.8%)	1
LEP history, *n* (%)		1 (8.3%)	8 (23.5%)	0.41
CIN grade	CIN1, *n* (%)	2 (16.7%)	24 (70.6%)	0.002
	CIN2, *n* (%)	10 (83.3%)	10 (29.4%)	
HPV type	high risk type, *n* (%)	6 (50.0%)	27 (79.4%)	0.14
	low risk type, *n* (%)	3 (25.0%)	4 (11.8%)	
Baseline CD86 expression (ΔCt) (median [IQR])		2.437 (1.799–3.866)	2.806 (1.544–3.546)	0.66
Change in CD86 expression (ΔCt diff) (median [IQR])		1.254 (0.047–2.371)	−0.980 (−3.034–0.420)	0.027

## Data Availability

The datasets generated and/or analyzed during the current study are not publicly available due to ethical and privacy restrictions related to patient data, but are available from the corresponding author on reasonable request and with appropriate institutional approvals.

## Supplementary Materials

The following supporting information can be downloaded at: https://www.mdpi.com/article/10.3390/biomedicines14071456/s1, Figure S1: Representative Papanicolaou-stained cervical cytology specimen. Figure S2: Representative images of negative control staining. Figure S3: Representative low- and high-magnification images of immunohistochemical staining. Table S1: Semi-quantitative assessment of immunohistochemical staining.

## Abbreviations

The following abbreviations are used in this manuscript:

CIN	Cervical intraepithelial neoplasia
HPV	Human papillomavirus
LBC	Liquid-based cytology
RT-qPCR	Reverse transcription–quantitative PCR
ROC	Receiver operating characteristic
OR	Odds ratio
HR	Hazard ratio
CI	Confidence intervals
