# Supplementary material for: Longitudinal Change in CD86 Expression Is Associated with Regression of Cervical Intraepithelial Neoplasia
Source: Biomedicines. 2026 Jun 26;14(7):1456. doi: 10.3390/biomedicines14071456 (PMC13405966; doi:10.3390/biomedicines14071456)
Supplement: Supplementary file 1 [file biomedicines-14-01456-s001.zip › biomedicines-4363420-supplementary.pdf]

Supplementary Material

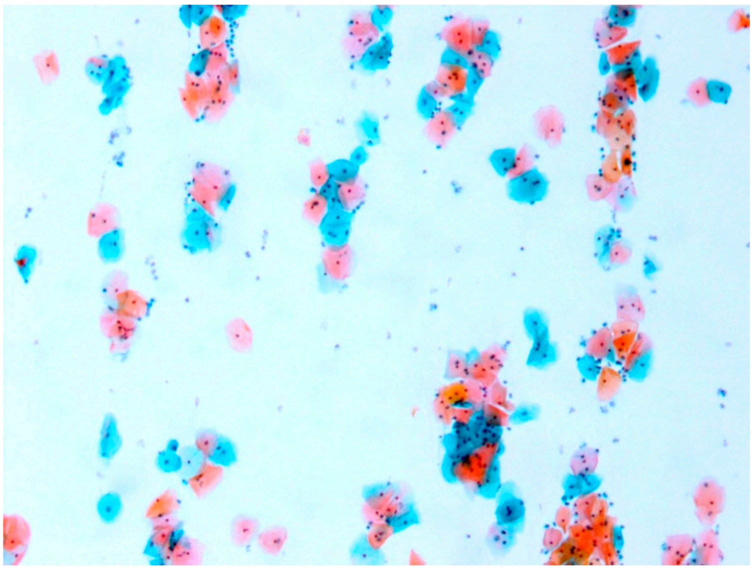

Figure S1. Representative Papanicolaou-stained cervical cytology specimen showing predominantly epithelial cells. Original magnification, ×8.

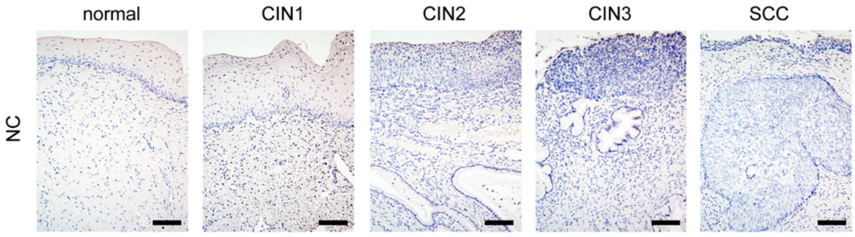

Figure S2. Representative images of negative control staining.

Representative negative control sections processed in parallel with the immunohistochemical staining protocol. Primary antibodies were omitted, and no specific staining was observed. Scale bars = 100 μm.

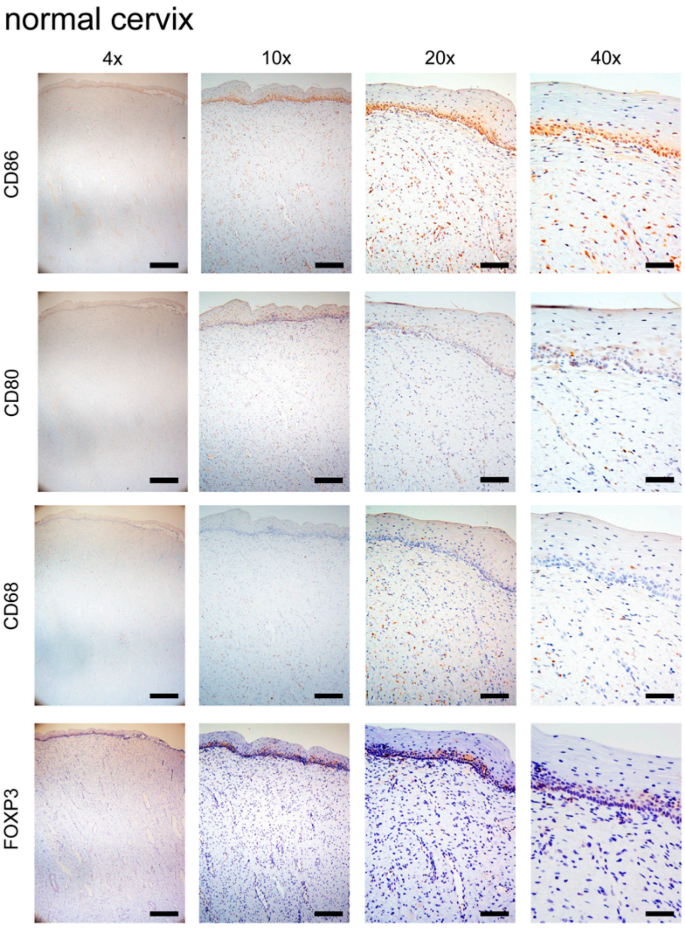
normal cervix
4x
10x
20x
40x
CD86
CD80
CD68
FOXP3

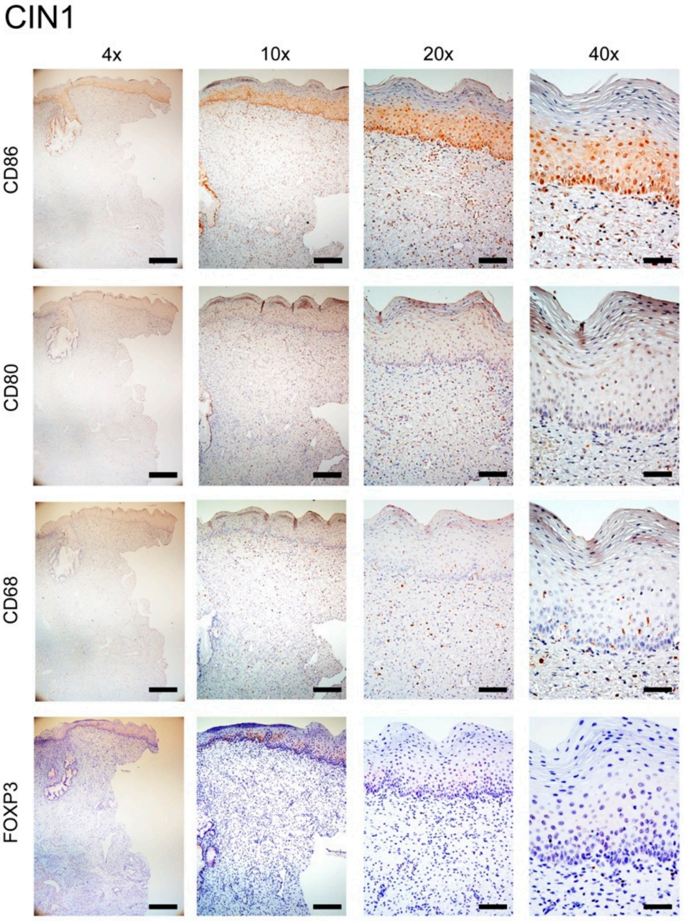
CIN1
4x
10x
20x
40x
CD86
CD80
CD68
FOXP3

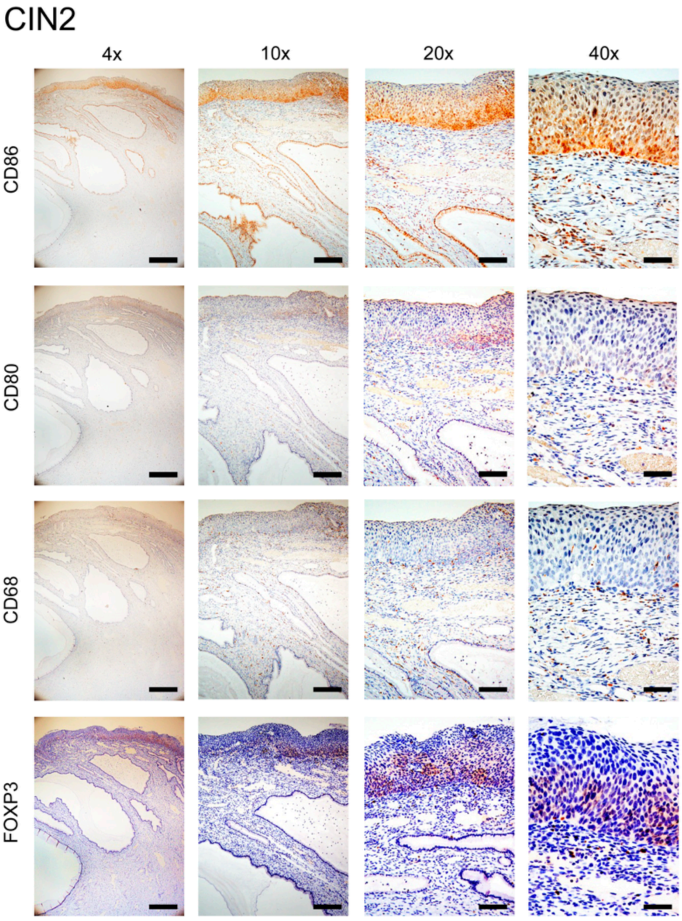
CIN2
4x
10x
20x
40x
CD86
CD80
CD68
FOXP3

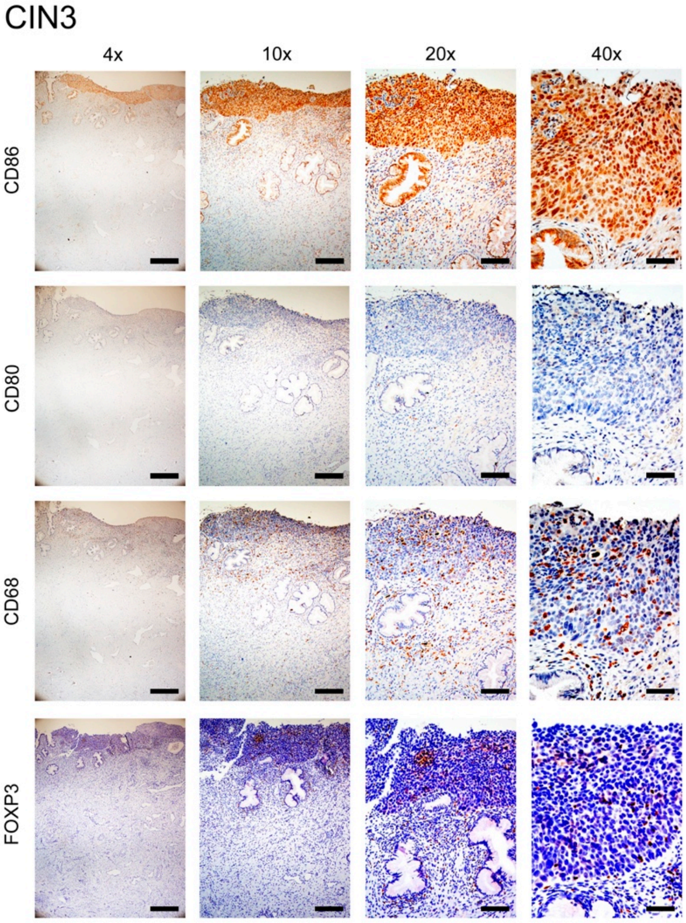
CIN3
4x
10x
20x
40x
CD86
CD80
CD68
FOXP3

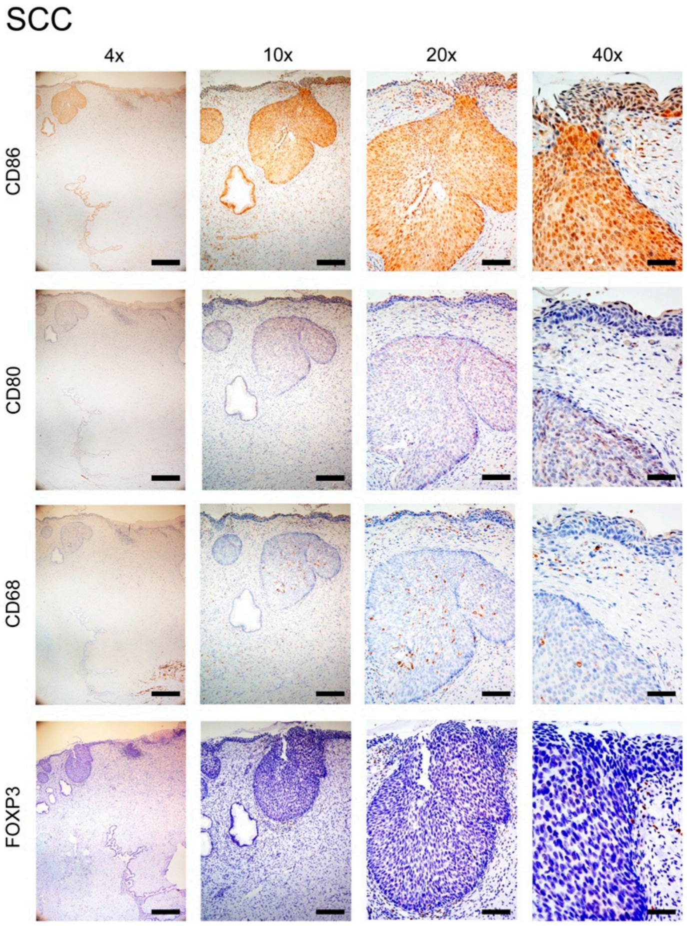

Figure S3. Representative low- and high-magnification images of immunohistochemical staining.

Representative low-power (4x or 10x) and high-power (20x or 40x) views of CD86, CD80, CD68, and FOXP3 staining in cervical tissues. The high-power images correspond to the boxed regions shown in the low-power views. Representative images are shown in the order of the normal cervix, CIN grades 1–3, and cervical squamous cell carcinoma (SCC) tissues. Scale bars are indicated in each panel. Scale bars = 500 μm, 200 μm, 100 μm, and 50 μm (4x, 10x, 20x, and 40x).

	CD86 H-score (median [range])	CD80 H-score (median [range])	FOXP3-positive cell ratio (percentage, median [range])
Normal cervix (n = 3)	147.4 (146.0–150.0)	19.7 (5.7–26.0)	0.27 (0–1.22)
CIN1 (n = 3)	148.5 (133.7–157.3)	17.4 (16.1–18.7)	0.52 (0.27–0.84)
CIN2 (n = 3)	256.8 (249.1–262.8)	21.7 (17.0–27.6)	2.73 (2.42–4.63)
CIN3 (n = 3)	291.8 (286.0–293.3)	3.4 (3.0–13.0)	4.65 (3.34–5.08)
SCC (n = 3)	226.8 (213.8–264.9)	5.0 (3.4–7.6)	2.21 (2.14–5.53)

Table S1. Semi-quantitative assessment of immunohistochemical staining.

Semi-quantitative H-score assessment of CD86 and CD80 staining in normal cervix, CIN1, CIN2, CIN3, and squamous cell carcinoma (SCC) tissues. FOXP3-positive cells were evaluated as the percentage of FOXP3-positive cells in the nucleus relative to basal epithelial cells. CD68 staining was assessed qualitatively to evaluate the localization of macrophage-lineage cells relative to CD86-positive epithelial staining and was not included in the H-score analysis. Data are presented as medians with interquartile ranges.
